# Case-Book Extracts

**Published:** 1837

**Authors:** 


### Art II.—Case-Book Extracts.

*Several short cases of Disease, extracted from the Note Book of* Dr. A. B. Price, of Centreville, Ohio.

### Case 1.—Temporary Parturient Blindness.

Mrs. B. was confined, 2nd child, January 5th, 1837. The labor at the commencement was attended with flooding, which ceased before the child was born or the membranes ruptured. After the birth of the child it returned, though the uterus contracted well; at this time she took some sugar of lead.

On the 8th day of January, or three days from the accouchment, about 11 A. M., she was seized with nearly total loss of *distinct* vision, which continued 4 hours. During the paroxysm there was great intolerance of light, so much so, that the room had to be kept darkened; no pain in the head or eyes; eyes natural aspect; pulse intermittent, this was the character of the pulse when confined; skin cool, extremities cold; bowels costive; lochia free; great mental agitation; when she closes her eyes frightful images haunt her imagination and prevent sleep. Prescription, 2 ounces castor oil.

9th. About the same hour, loss of vision returned, attended with considerable pain and throbbing in the head; extremities cool; pupil contracted; thirst; pulse as yesterday. Blister to the nape of the neck, and ordered a table spoon full of infusion of valerian every two hours. Evening visit found she had been in a warm sweat soon after commencing the infusion; blister drawn well; less pain and throbbing in the head, can see better. Gave calomel 20 grs, pulv. antimonialis 5 grs., cold applications to the head.

10th. Morning. Sight returned at dusk yesterday, and remains good, no pain, pulse regular and eyes natural, rested well, calomel operated twice.

Evening. Sight less perfect, complains of every thing being in motion, slight pain in the head, blister discharges freely, calomel repeated.

11th. Better; calomel operated well; skin moist; ordered quinine; continued to improve from this time. This lady was affected in the same way when her first child was born. It then continued about two weeks.

### Case II.—Miasmatic Neuralgia.

June 1st, 1837, I was called to prescribe for a lad aged about 12 years. Has had the ague this spring, for which he took quinine; for the last two weeks has complained of a severe pain in the right eye, from about 8 to 11 A. M. every day, preceded by a sense of coldness and followed by slight fever. Eyes look natural; anorexia; tongue furred, brown. The pain in the eye commenced in a few days after the ague left him.

Prescribed an antimonial emetic, and after the operation of it, while free from pain, an acidulated solution of quinine every two hours during the intermission. This course immediately suspended the paroxysms.

Here I would remark, that such affections are not uncommon in districts of country where the ague prevails. In the course of a few years I have seen many cases. It occurs generally in those who have had that disease, and comes on soon after the apparent cessation of it. In all the cases which have come under my notice, there was gastric and biliary derangement, evinced by anorexia, furred tongue, bitter taste in the mouth, &c. Considering it to be essentially intermittent fever, I have uniformly prescribed for it as I would for that disease—and always with success.

There is a similar affection vulgarly called "sun pain," from the circumstance that it comes on as the sun rises, and gradually increases until noon, then declines and finally disappears as the sun sinks beneath the horizon, to be repeated the next day. This also is easily cured by first evacuating the stomach and bowels during the paroxysm, and giving large doses of quinine at night. In this affection I have seen nothing give such immediate but temporary relief, as Carb. Ammonia 10 grains, Pulv. Cayenne 5 to 10. This composition will act like a charm in removing the pain.

### Case III.—Temporary Aphonia.

June 8, was called to see a little girl, aged about eight years, laboring under aphonia, produced by the practice of whirling round swiftly while at school. It was complete, and continued about 12 hours. There was nothing to lead a person to suspect any thing of its nature. In fact, there was no discoverable alteration from health, except the loss of voice, and a pulse rather slower than natural, and small; no heat of the head; perfect use of her limbs and tongue; eye natural; no pain or uneasiness of any kind. Not knowing the cause at the time, and suspecting that worms might be present, I gave her calomel, to be followed by castor oil and turpentine.

A few days after, another of the same school was taken in the same way, but in a few hours entirely recovered. It occurred in both soon after having practised whirling round.

While on this subject, I will mention the case of a young lady of this vicinity, who has labored under aphonia for nearly two years, and in whom there is no deviation from health, nor was there for some time before the attack. She cannot speak louder than a soft whisper, yet she can sing loud, and with perfect ease. When she makes an effort to converse, it appears to require all the muscular strength she is master of, and she cannot then produce an audible sound, only as she inhales the air.

### Case IV.—Poisoning by the fruit of the Prunus Virginiana.

The following case, with many others, occurred in the fall of 1834. That year, it will be recollected, was remarkable for the scarcity of cultivated fruit. But there was a great quantity of wild cherries, of which children generally partook plentifully. A number of cases similar to this occurred, but one is sufficient to detail for the present.

Sept. 4, 1834, was called at 9 P. M. to see a child of Mr. S. In the afternoon had eat a quantity of wild cherries, which produced the following class of symptoms: Vomiting, stupor, pupil dilated and insensible to light, loss of muscular strength, pulse small and frequent, breathing slow, skin pale, jaws clenched, inability to swallow or speak. After the stomach was unloaded by vomiting, nausea and constant efforts to vomit continued. The retching brought up a tough, glazy mucous. Extremities cold.

Treatment.—Pressed open the jaws and gave her aqua ammonia. Sinapisms to the stomach and extremities. Injections of sulph. mag.; gave three before they were returned, the last of which was combined with table salt. Soon after this was administered she became restless, and had a copious evacuation of the bowels. The vomiting ceased, and in a short time she became conscious. Nothing more was done than to administer some castor oil.

I believe that it is not generally known that the fruit of the wild cherry is poisonous,* but such is the fact, as the above case sufficiently proves. Many kinds of birds are intoxicated by the fruit, and easily caught. It would seem that they produced narcotic symptoms; perhaps a valuable medicine might be obtained from them. Physicians should caution parents in regard to this fruit.

July 14th, 1837.

